# Supplementary material for: From sequence to enzyme mechanism using multi-label machine learning
Source: BMC Bioinformatics. 2014 May 19;15:150. doi: 10.1186/1471-2105-15-150 (PMC4229970; doi:10.1186/1471-2105-15-150)
Supplement: Additional file 2 — Java code of ml2db. Additional file ml2db_code.tar.gz contains the Java source code to run the multi-label machine learning experiments and save the results to database. The code’s Javadoc is included. [file 1471-2105-15-150-S2.zip › additional file 2/ml2db/ml2db/doc/mulan/classifier/lazy/BRkNN.ExtensionType.html]

BRkNN.ExtensionType


JavaScript is disabled on your browser.


- Overview
- Package
- Class
- Use
- Tree
- Deprecated
- Index
- Help

- Prev Class
- Next Class

- Frames
- No Frames

- All Classes

- Summary:
- Nested |
- Enum Constants |
- Field |
- Method

- Detail:
- Enum Constants |
- Field |
- Method


mulan.classifier.lazy

## Enum BRkNN.ExtensionType

- java.lang.Object
- - java.lang.Enum<BRkNN.ExtensionType>
  - - mulan.classifier.lazy.BRkNN.ExtensionType

- All Implemented Interfaces:
  :   java.io.Serializable, java.lang.Comparable<BRkNN.ExtensionType>

  Enclosing class:
  :   BRkNN

  ---

    

  ```
  public static enum BRkNN.ExtensionType
  extends java.lang.Enum<BRkNN.ExtensionType>
  ```

  The two types of extensions

- - ### Enum Constant Summary

    Enum Constants

    Enum Constant and Description

    `EXTA`

    Predict top ranked label in case of empty prediction set

    `EXTB`

    Predict top n ranked labels based on size of labelset in neighbors

    `NONE`

    Standard BR
  - ### Method Summary

    Methods

    | Modifier and Type | Method and Description |
    | --- | --- |
    | `static BRkNN.ExtensionType` | `valueOf(java.lang.String name)` Returns the enum constant of this type with the specified name. |
    | `static BRkNN.ExtensionType[]` | `values()` Returns an array containing the constants of this enum type, in the order they are declared. |

    - ### Methods inherited from class java.lang.Enum

      `compareTo, equals, getDeclaringClass, hashCode, name, ordinal, toString, valueOf`
    - ### Methods inherited from class java.lang.Object

      `getClass, notify, notifyAll, wait, wait, wait`

- - ### Enum Constant Detail


    - #### NONE

      ```
      public static final BRkNN.ExtensionType NONE
      ```

      Standard BR


    - #### EXTA

      ```
      public static final BRkNN.ExtensionType EXTA
      ```

      Predict top ranked label in case of empty prediction set


    - #### EXTB

      ```
      public static final BRkNN.ExtensionType EXTB
      ```

      Predict top n ranked labels based on size of labelset in neighbors
  - ### Method Detail


    - #### values

      ```
      public static BRkNN.ExtensionType[] values()
      ```

      Returns an array containing the constants of this enum type, in
      the order they are declared. This method may be used to iterate
      over the constants as follows:

      ```
      for (BRkNN.ExtensionType c : BRkNN.ExtensionType.values())
          System.out.println(c);
      ```

      Returns:
      :   an array containing the constants of this enum type, in
          the order they are declared


    - #### valueOf

      ```
      public static BRkNN.ExtensionType valueOf(java.lang.String name)
      ```

      Returns the enum constant of this type with the specified name.
      The string must match *exactly* an identifier used to declare an
      enum constant in this type. (Extraneous whitespace characters are
      not permitted.)

      Parameters:
      :   `name` - the name of the enum constant to be returned.

      Returns:
      :   the enum constant with the specified name

      Throws:
      :   `java.lang.IllegalArgumentException` - if this enum type has no constant
          with the specified name
      :   `java.lang.NullPointerException` - if the argument is null


- Overview
- Package
- Class
- Use
- Tree
- Deprecated
- Index
- Help

- Prev Class
- Next Class

- Frames
- No Frames

- All Classes

- Summary:
- Nested |
- Enum Constants |
- Field |
- Method

- Detail:
- Enum Constants |
- Field |
- Method
